# Avian fitness consequences match habitat selection at the nest‐site and landscape scale in agriculturally fragmented landscapes

**DOI:** 10.1002/ece3.5288

**Published:** 2019-06-11

**Authors:** Bryan M. Reiley, Thomas J. Benson

**Affiliations:** ^1^ Illinois Natural History Survey Prairie Research Institute University of Illinois Champaign Illinois; ^2^ Department of Natural Resources and Environmental Sciences University of Illinois Champaign Illinois

**Keywords:** Bell's Vireo, fitness, habitat selection, landscape, nest site, patch size, Willow Flycatcher

## Abstract

1. Habitat selection theory suggests that when choosing breeding sites, animals should choose the best available habitat; however, studies show that individuals fail to choose habitats that maximize their fitness especially in drastically altered landscapes. Many studies have focused on selection at single scales, often using a single measure of fitness. However, links between habitat selection and fitness may vary depending on the spatial scale and measure of fitness, especially in situations where agricultural land use has altered the surrounding landscape.
2. We examined multiscale habitat selection and fitness measures of the Bell's Vireo (*Vireo bellii*) and Willow Flycatcher (*Empidonax traillii*) using data collected in agriculturally fragmented landscapes.
3. We found evidence for selection of nest sites with dense understory, larger patches, and increasing restored habitat cover and decreasing forest cover in the surrounding landscape.
4. For both focal species, selection for dense understory significantly increased nest survival; however, there appeared to be no concurrent benefit for fledgling production. Selection for broader scale features positively influenced nest survival for the Willow Flycatcher with no concurrent benefit for fledgling production. The observed mismatches may be due to anthropogenic habitat fragmentation at broader scales or may represent reproductive trade‐offs for a fitness benefit not measured in this study.
5. Fine‐scale habitat selection decisions by our focal species appear to match fitness outcomes, whereas habitat selection at broader scales only provided fitness benefits for the Willow Flycatcher. Though providing no fledgling production benefit, when combined with suitably dense nesting habitat, larger patches in landscapes with greater amounts of restored habitat cover for Bell's Vireo and lower amounts of forest cover for Willow Flycatcher will produce more fledglings per unit area than smaller patches in landscapes with less restored habitat and more forest cover, respectively, which could help inform conservation decisions for these at‐risk species.

## 1 INTRODUCTION

Understanding why animals choose habitat is important for understanding how they assess habitat quality (i.e., fitness potential). This is especially true for migratory species whose mobility provides access to an array of potential habitat patches over vast spatial scales. For migratory individuals, choosing where to breed has important reproductive implications (Holmes, Marra, & Sherry, 1996; Kolbe & Janzen, 2002; Misenhelter & Rotenberry, 2000) as patches differ in potential quality, and habitat quality within patches is rarely homogeneous. Natural selection should favor the ability to assess habitat quality (Jaenike & Holt, 1991; Martin, 1998) and animals should choose the best available habitat (Fretwell & Lucas, 1970). Consequently, we would expect clear links between habitat selection and resulting fitness consequences.

Despite the expected links between habitat selection and fitness outcomes, individuals often fail to choose habitats that maximize their fitness potential (Chalfoun & Martin, 2007; Germain & Arcese, 2014; Woodward, Fink, & Thompson, 2001). While studies have found positive relationships between habitat selection (i.e., individuals select one habitat feature over unused but available habitat features) and fitness outcomes (Martin, 1998; McKellar, Kesler, & Walters, 2015; Perot & Villard, 2009), many studies have documented mismatches between selection and fitness (i.e., either equally selecting or preferring low‐ over high‐quality habitat; Arlt & Pärt, 2007; Mägi et al., 2009). Explanations for mismatches between habitat selection and fitness outcomes have been varied and include lack of available cues at the time of selection (Orians & Wittenberger, 1991), selection cues not linked to habitat quality (Schlaepfer, Runge, & Sherman, 2002), conflicting choices between other cues and mate choice (Kokko & Sutherland, 2001), and site fidelity (Pulliam & Danielson, 1991). Maladaptive habitat selection has frequently been explained to result from anthropogenic disturbance whereby traditional indicators of habitat quality become unreliable (Bock & Jones, 2004; Misenhelter & Rotenberry, 2000; Weldon & Haddad, 2005).

To accurately estimate reproductive consequences of habitat selection decisions, studies must focus on biologically relevant spatial scales (Chalfoun & Martin, 2007). As a result of predation being the primary cause of failure for songbird nests (Ibáñez‐Álamo et al., 2015; Martin, 1993; Ricklefs, 1969), evaluations of adaptive habitat selection for these species have often focused on the influence of habitat structure immediately surrounding nests on predation (Chalfoun & Schmidt, 2012; Martin, 1998; Misenhelter & Rotenberry, 2000, but see Sperry, Peak, Cimprich, & Weatherhead, 2008). At this scale, habitat features such as understory density can influence fitness outcomes by affecting the probability of nest discovery by predators (Martin, 1998), and as a result, birds may choose breeding locations with thicker understory vegetation to minimize the risk of predation (Martin, 1993). At the nest site, microclimate may also influence the number of fledglings produced per successful nest (Lloyd & Martin, 2005). Beyond the nest site, broadscale features may influence the distribution and abundance of predators and brood parasites. For example, nests in smaller patches may be more vulnerable to generalist nest predators (Rush & Stutchbury, 2008) and may face a greater threat of parasitism by Brown‐headed Cowbirds (*Molothrus ater)* (Benson, Chiavacci, & Ward, 2013; Hoover, Brittingham, & Goodrich, 1995; Rush & Stutchbury, 2008). Additionally, the number of young fledged from successful nests can be affected by food abundance and habitat features that affect foraging efficiency (Pärt, 2001), both of which may be influenced by attributes of the patch where they breed. Moreover, nest predation is spatially heterogeneous and dependent on landscape context (Tewksbury et al., 2006). For example, nests located in highly fragmented and agriculturally dominated landscapes may have greater predation and brood parasitism rates than nests in more intact landscapes (Chalfoun, Thompson, & Ratnswamy, 2002; Donovan, Thompson, Faaborg, & Probst, 1995).

Multiscale studies of adaptive habitat selection that document both habitat selection and resulting components of reproductive performance are rare (Clark & Shutler, 1999; Lloyd & Martin, 2005; Martin, 1998), and this type of research is particularly needed in habitats embedded in agriculturally fragmented landscapes where there is a high potential for mismatches between selection and reproductive consequences (Gilroy, Anderson, Vickery, Grice, & Sutherland, 2011; Weldon & Haddad, 2005). Restored habitats in agricultural landscapes, while widely thought to be beneficial for birds (Herkert, 2007), have also been suggested to have fecundity rates too low to sustain local populations of some species (Fletcher, Koford, & Seaman, 2006; McCoy, Ryan, Kurzejeski, & Burger, 1999). Thus, understanding habitat choice and reproductive outcomes in these restored habitats could both provide new insights into the adaptive nature of habitat selection in birds and provide managers with valuable information to be applied to future conservation efforts for priority species.

Here, we studied the breeding habitat selection and two reproductive metrics (nest success and fledgling production) of two species of conservation concern. We did this across three scales that we assumed to be ecologically relevant for our focal bird species: (a) nest site (5‐m buffer around nest), (b) landscape (1,200‐m buffer), and (c) habitat patch using data collected in restored habitat on former farmland where we expected a high potential for mismatches between habitat selection and fitness consequences. Specifically, based on previous work (Joos, Thompson, & Faaborg, 2014; Kus, Hopp, Johnson, & Brown, 2010; Reiley & Benson, 2019; Sedgwick, 2000) we expected habitat selection for both species at the nest‐site scale would be related to habitat features associated with increased cover at the nest, both species would be associated with increased patch size, would be positively associated with surrounding grassland cover. Relative to fitness consequences of habitat selection, given that previous studies (e.g., Chalfoun & Schmidt, 2012 and references therein) found the highest probability of finding evidence for adaptive habitat selection was at the nest scale and long‐standing associations between avian species and vegetation around the nest site, we expected that nest‐site habitat selection for both species would result in greater nest survival and fledgling production. Conversely, because the alteration of landscapes and creation of patchy landscapes due to agricultural fragmentation is relatively novel, as measured on evolutionary timescales, we expected that habitat selection for patch and landscape features should lead to either neutral or maladaptive selection.

## 2 MATERIALS AND METHODS

### 2.1 Study system

Our focal species were the Bell's Vireo (*Vireo bellii bellii*) (hereafter vireo) and Willow Flycatcher (*Empidonax traillii traillii*) (hereafter flycatcher), both of which are species of conservation concern due to recent declines. Both species are single‐brooded, small, insectivorous Neotropical migrants that breed in dense understory vegetation associated with shrubland habitats (Kus et al., 2010; Sedgwick, 2000).

Data collection took place in Illinois, USA (40°15′N, −90°28′), in six counties (Christian, Fulton, Logan, McDonough Sangamon, and Schuyler). Sample fields (*n* = 172) were randomly drawn from a group of former agricultural fields restored through a farmland restoration program. Sample fields ranged from 2.9 to 174.7 ha ($\bar{x}$ = 35.3, *SE* = 2.6). Fields were dominated by early‐successional vegetative communities (restoration efforts began in 1999). Fields were frequently adjacent to riparian forests and row‐crop agriculture.

### 2.2 Habitat selection

To determine vireo and flycatcher patch and landscape habitat selection, fields were sampled using standard point‐count methods during the breeding seasons of 2012–2015. Surveys consisted of unlimited‐radius point counts, 10 min in duration (Ralph, Sauer, & Droege, 1993). For specific details regarding point‐count methods, see Appendix S1. To examine habitat use at the nest scale and study the reproductive consequences of habitat selection, we searched for nests at a subset of our sample fields. Nest‐sampling fields were chosen from among the point‐count fields based on the presence of our focal species (from point‐count surveys) and through the use of additional survey transects to identify territories of focal species (for more details, see Appendix S1).

### 2.3 Fitness consequences

Nest searches were conducted from May–August 2013–2015 at 14 focal fields, 7 had only vireos, 2 had only flycatchers, and 5 had both species. Focal fields for nest searching ranged from 5.4 to 81.4 ha ($\bar{x}$ = 28.9 ha) and were separated by >4 km. We searched for all vireo and flycatcher nests in each focal field systematically (walking a grid within occupied areas) and by using behavioral cues. Nest searches were conducted in all areas identified as occupied every 1–3 days until an active nest was found. Areas where a nest was deemed inactive or failed were subsequently searched to monitor additional nesting attempts for each pair. While we did not band individuals, we felt confident our renest searches were focused on the same territory due to the relatively wide spacing of males (Kus, et al., 2010; Sedgwick, 2000) as well as finding few inactive (<3% of total nests per year) nests during subsequent systematic nest searches.

### 2.4 Nest scale variables

After the termination of nesting each year, we recorded data on vegetation structure at both nest sites (around July 15) and a paired random location within a 160‐m radius of each nest site using a modified BBIRD protocol (Martin et al., 1997). We chose this radius based on average spacing of males at our study sites (±200 m) and based on the small territory size of our focal species (Kus et al., 2010; Sedgwick, 2000), we felt like a 160‐m radius around a nest would prevent overlapping an adjacent male's territory and provide enough area to characterize what habitat was available. Habitat variables were chosen based on previous studies or presumed relevance to the nesting ecology of early‐successional birds and search efficiency of predators (see Appendix S1).

### 2.5 Patch and landscape scale variables

At both the patch and landscape scales, we used avian density derived from point‐count data as a measure of habitat selection of our focal species. Variables were chosen based on previous studies, presumed relevance to the nesting ecology of early‐successional birds and the predators that affect them. Specifically, we quantified patch size (ha) of each field where point counts occurred and where we searched for nests by measuring all continuous habitat, considering roads with two or more lanes with disturbed roadsides or habitat transitions (i.e., grassy field to forest interface), and we quantified landscape composition (i.e., grassland, forest, and restored habitat) within a 1,200‐m buffer around each field used for habitat selection and each nest used for nest survival and fledgling analyses (for details, see Appendix S1).

### 2.6 Reproductive consequences

We examined the influence of nest‐site, patch, and landscape variables on two measures of avian fitness: daily nest survival (Shaffer, 2004) and the number of young fledged per successful nest. Nest survival helps differentiate complete failure from nests that fledged at least one young, whereas the number fledged from successful nests can help determine the influence of factors other than predation (e.g., microclimate and food availability).

### 2.7 Statistical analyses

To determine vireo and flycatcher nest‐site selection, we used generalized linear mixed models with a binomial distribution and logit link function (SAS PROC GLIMMIX; SAS Institute, 2010). Due to the proximity of nests and random locations and because year may have an effect on vegetation variables, we considered models with nest and year as random effects. In this analysis, nest presence was the response variable and understory density, grass cover, forb cover, and shrub cover were predictor variables (for a detailed modeling description, see Appendix S1).

To determine vireo and flycatcher patch and landscape habitat selection (point‐count data), we used the extended hierarchical distance sampling model of Royle, Dawson, and Bates (2004) using the gdistsamp function in the unmarked package in R (Fiske & Chandler, 2011) which can be used to model site‐scale covariates for both detection probability and density. Specifically, we used this function to evaluate models that describe how abundance varied as functions of covariates at patch and landscape spatial scales. For the abundance portion of the model, spatial variation in the number of birds (primarily singing males) was treated as a negative binomial random variable. To account for the sampling of each site in four separate years, we included year as a covariate for abundance in each model. For further distance sampling analysis description, see Appendix S1.

To evaluate patch and landscape selection, we developed two sets of a priori models, one for patch scale and another for landscape scale (Table 1). We ranked models according to AIC_c_ and computed model weights (Burnham & Anderson, 2002). In cases where no model was overwhelmingly supported (*w_i_* > 0.9), we used model averaging using the natural average method in the R package AICcmodavg (Mazerolle, 2012) to examine effects of explanatory variables on bird abundance. For a detailed modeling description, see Appendix S1.

**Table 1:** Model‐averaged parameter estimates (*β*) and 95% confidence limits for best fit nest‐site, patch, and landscape habitat selection variables for Bell's Vireo and Willow Flycatcher in restored farmland habitats in Western Illinois, USA, 2012–2015

Parameters	Bell's Vireo			Willow Flycatcher		
	*β*	LCL	UCL	*β*	LCL	UCL
Nest‐site selection						
% Understory density	0.006	0.002	0.010	0.011	−0.001	0.023
Landscape selection						
% Forest within 1,200 m				−0.25	−0.37	−0.13
% Restored habitat within 1,200 m	0.23	0.07	0.39			
Patch selection						
Patch size	0.200	0.030	0.380	0.030	−0.080	0.150

To understand whether habitat selection was indicative of habitat quality at the nest‐site, patch, and landscape scale, we examined vireo and flycatcher nest survival using the logistic exposure method (Shaffer, 2004) and the number of young fledged from successful nests using generalized linear mixed models using Poisson distribution (SAS PROC GLIMMIX; SAS Institute, 2010). To account for potential nonindependence due to repeated sampling of sites within and among years, we included year and site as random effects but later dropped these random effects as they did not improve models. We evaluated nest survival by generating four sets of models: temporal and biological, nest‐site, patch, and landscape. Although not to the focus of our study, we dealt with potential nuisance variables by including temporal and biological models for nest survival including year, day of year, year × day of year, parasitism status, nest stage × year, and nest stage × day of year + year (nest survival only). Because temporal and biological factors reflect important sources of variation that may affect the influence of habitat or landscape features, we evaluated these models first using AIC_c_ and included the best fit variables in subsequent nest survival and fledgling production analyses. For both nest survival and fledgling production analyses, we tested our predictions using a final model for each scale including the best fit nuisance variable along with the best fit nest‐site, patch, and landscape variables and generated model coefficients using that model. As above, we assessed support for fitness outcomes relative to species habitat selection using model coefficients and their 95% confidence intervals. We considered variables that were supported by AIC_c_, but for which confidence intervals of coefficients that overlapped zero to be weakly supported.

## 3 RESULTS

From 2013 to 2015, we found 572 vireo nests (505 with eggs or young) and 204 flycatcher nests (188 with eggs or young). Of these, 174 (34%) vireo and 106 (56%) flycatcher nests fledged host young ($\bar{x}$ = 2.89, *SE* = 0.08, $\bar{x}$ = 2.86, *SE* = 0.09, young per successful nest, respectively). We collected habitat data at paired nest‐site and random locations for 572 vireo nests and 132 flycatcher nests. Out of the 505 active vireo nests, we had complete nest‐site habitat, patch, and landscape composition data for 466 nests for 1,808 exposure days and included those in subsequent nest survival analyses. We had complete nest‐site habitat, patch, and landscape composition data for all active flycatcher nests for 746 exposure days and included those in subsequent survival analysis. Brown‐headed Cowbird parasitism rates were 31.4% (*n* = 159, 8 fledged young and 146 were abandoned) for vireo nests and 16.5% (*n* = 31) for flycatcher nests.

### 3.1 Habitat selection

At the nest‐site scale (nest data), both vireos and flycatchers selected nest sites with greater density of understory vegetation ($\bar{x}$ = 0.81, *SE* = 0.003) compared with unused but available surrounding vegetation ($\bar{x}$ = 0.49, *SE* = 0.02) (Table 1 and see Table S1). At the patch scale (point‐count data), vireo density was greater in larger patches ($\bar{x}$ = 25.3 ha, *SE* = 6.2) with this variable appearing in models with combined AIC_c_ weight of 60% (Table 1 and see Table S2) and we found no influence of patch size on habitat use by flycatchers (Table 1). At the landscape scale (point‐count data), density of vireos was greater with increasing amounts of restored habitat and density of flycatchers significantly declined with increasing forest cover within 1,200 m with these variables appearing in models with combined AIC_c_ weight of 89% and 99%, respectively (Table 1 and see Table S2).

### 3.2 Nest survival

**Figure 1:**
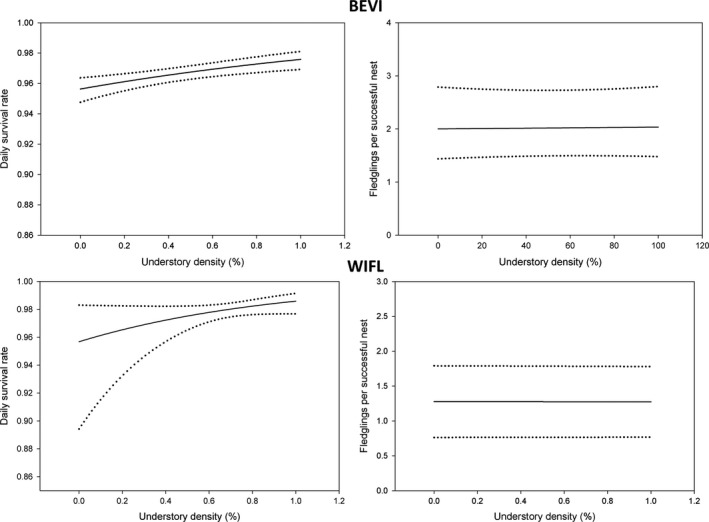
Predicted daily survival rate and numbers of fledglings produced per successful nest (with 95% confidence intervals) for Bell's Vireo (BEVI) and Willow Flycatcher (WIFL) nests relative to % understory density within 5 m of a nest

**Figure 2:**
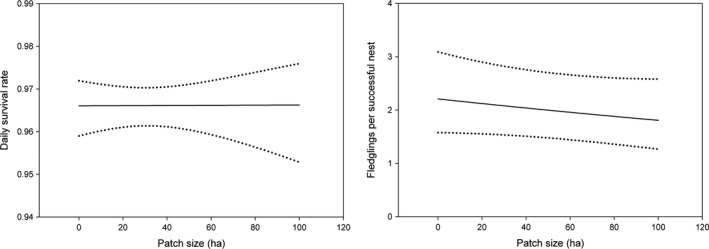
Predicted daily survival rate and numbers of fledglings produced per nest and 95% confidence intervals of Bell's Vireo nests relative to patch size (ha)

**Figure 3:**
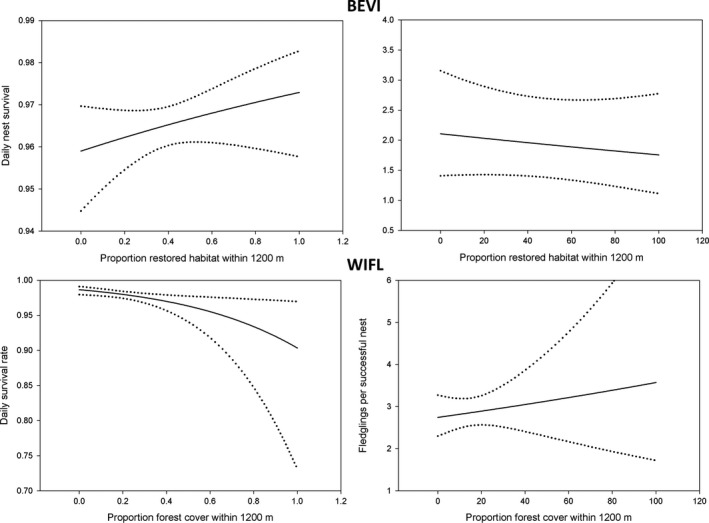
Predicted daily survival rate and numbers of fledglings produced per nest and 95% confidence intervals of Bell's Vireo (BEVI) and Willow Flycatcher (WIFL) nests relative to % restored habitat within 1,200 m and % forest cover within 1,200 m of a nest, respectively

The best fitting temporal and biological model for vireo nest survival incorporated a negative effect of parasitism status (Table 2 and see Table S3), and the estimated daily nest survival of parasitized nests was 0.923, *SE* = 0.01 (0.16 probability of surviving to fledging; assuming 24‐day nesting period), and that of nonparasitized nests was 0.965, *SE* = 0.003 (0.44 probability of surviving to fledging; assuming a 24‐day nesting period). Daily survival rate of vireo nests was positively related to understory density (Figure 1; Tables 2 and 3 and see Table S4). There was no relationship between nest survival and patch size (Figure 2; Tables 2 and 3). For Bell's Vireo, nest survival was positively associated with the proportion of restored habitat within 1,200 m (Figure 3); however, the 95% confidence interval of the coefficient overlapped zero (Tables 2 and 3).

**Table 2:** Model‐averaged parameter estimates (*β*) and 95% confidence limits, for best fit biological and habitat variables from logistic exposure models of nest survival and number of young fledged from successful nests (fledgling production) from Bell's Vireo and Willow Flycatcher nests at restored farmland habitats in Western Illinois, USA, 2012–2015

Parameters	Nest survival						Fledgling production					
	Bell's Vireo			Willow Flycatcher			Bell's Vireo			Willow Flycatcher		
	*β*	LCL	UCL	*β*	LCL	UCL	*β*	LCL	UCL	*β*	LCL	UCL
Temporal and biological models												
Intercept				3.980	3.725	4.235				1.049	0.936	1.163
Parasitism^a^	−0.770	−1.358	−0.182				0.769	0.181	1.357			
Habitat models												
% Understory density	0.072	0.064	0.079	0.072	0.020	0.380	0.000	−0.002	0.003	−0.003	−0.010	0.004
Patch size	0.000	−0.004	0.004				−0.002	−0.005	0.001			
% Forest within 1,200 m				−2.06	−3.61	−0.514				0.263	−0.582	1.113
% Restored habitat within 1,200 m	0.429	−0.294	1.152				−0.1823	−0.357	0.723			

a Probability of Brown‐headed Cowbird parasitism.

**Table 3:** Summary of results from models of nest survival and fledgling production comparing habitat selection and fitness outcomes from Bell's Vireo and Willow Flycatcher nests at restored farmland habitats in Western Illinois, USA, 2012–2015

	Bell's Vireo		Willow Flycatcher	
	Nest survival	Fledgling production	Nest survival	Fledgling production
Understory density	*Strong match*	Neutral	*Strong match*	Neutral
Patch size	Neutral	Weak mismatch		
% Forest within 200 m			*Strong match*	Weak mismatch
% Restored habitat within 1,200 m	Weak match	Weak mismatch		

No temporal or biological model for flycatcher nest survival fit better than the constant‐survival model (see Table S3) with an estimated daily nest survival of 0.980, *SE* = 0.003 (0.63 probability of surviving to fledging; assuming 24‐day nesting period). At the nest scale, flycatcher daily nest survival rate was positively associated with understory density (Figure 1; Tables 2 and 3). Nest survival for flycatchers was greatest in nests with (Figure 3; Tables 2 and 3) less forest cover within 1,200 m.

### 3.3 Fledgling production

The best fitting temporal or biological model for the number of vireos fledging from successful nests incorporated a negative effect of parasitism status (Table 2 and see Table S4). The mean number of vireo fledglings for unparasitized nests (*n* = 166) was 2.97 (*SE* = 0.08) and 1.38 (*SE* = 0.42) fledglings per parasitized nest (*n* = 8). There was a negative relationship between fledgling production per nest and understory density (Figure 1); however, confidence intervals overlapped zero (Tables 2 and 3). Patch size and the amount of restored habitat within 1,200 m were both negatively associated with fledgling production (Figures 2 and 3); however, the relationship was weak (Tables 2 and 3).

The number of flycatchers fledged per successful nest decreased as understory density increased (Figure 3; Tables 2 and 3) and production was greater as the amount of forest habitat increased in the surrounding landscape (Figure 3); however, the relationships were weak (Tables 2 and 3).

## 4 DISCUSSION

We found clear but differing breeding habitat selection by our two focal species at all spatial scales examined. Despite breeding in agriculturally fragmented landscapes, we did not find that either species was selecting habitat in a way that led to decreased reproductive success. Importantly, our analyses demonstrated that the choice of increased understory at nest sites for both species resulted in greater nest survival but that fledgling production results were equivocal. For flycatchers, selection for reduced forest cover in the surrounding landscape increased nest survival; however, the remaining results showed no strong relationships between selection for patch or landscape features and measures of fitness.

Evidence for adaptive habitat selection provides support for the theoretical model, suggesting that animals should possess the ability to accurately assess habitat quality (Fretwell & Lucas, 1970). In birds, examples of adaptive selection have often been found at the nest‐site scale (i.e., Chalfoun & Schmidt, 2012). Likewise, we found higher fitness was associated with habitat selection at the nest‐site scale for our focal species. The most parsimonious explanation for this may be that predators are exerting selection pressure during this phase of the avian life cycle. Indeed, nest predation is the most important cause of avian nest failure (Chiavacci, Benson, & Ward, 2018; Ibáñez‐Álamo et al., 2015; Ricklefs, 1969). In support of this, after placing nest cameras at select nests (*n* = 47) in 2015, we found that 80% of known nest failures were caused by predation, with 55% due to depredation by snakes, avian predators, and mammals. Nest predation rates have been found to be influenced by nest‐site vegetation (Chalfoun & Martin, 2007; Martin, 1993, 1998) as well as nest height (Chiavacci et al., 2018), with generally reduced depredation rates with more concealment and higher nests. Additionally, concealment also influenced parasitism rates especially for Bell's Vireo which had a 28% decrease in nest success when parasitized. While high parasitism rates are common for this species (Budnik, Ryan, & Thompson, 2000; Kus et al., 2010) and they often abandon parasitized nests and renest to avoid fledging cowbird young (Kosciuch, Parker, & Sandercock, 2006), we found reduced parasitism rates with increased understory density (B. Reiley, unpublished), suggesting that nest concealment is not only important for influencing predation but also parasitism rates.

Support for adaptive habitat selection beyond the nest scale is rare (Chalfoun & Martin, 2007; Uboni, Smith, Stahler, & Vucetich, 2017; but see Joos et al., 2014; Kosterman, Squires, Holbrook, Pletsher, & Hebblewhite, 2018), with typical explanations including alternative fitness components not measured (i.e., Desare et al., 2014) or anthropogenic disturbance altering traditional fitness cues (i.e., DeCesare, 2012; Weldon and Haddad, 2005). Yet, we found that flycatcher selection for reduced forest cover within 1,200 m showed a clear nest survival benefit. This is perhaps not surprising given flycatchers’ habitat selection for nesting in riparian forest edges and studies of the nesting ecology studies of this species suggest that mammalian and avian predators are the most important (Sedgwick, 2000) which tend to be associated with forest edge habitat (Cain, Morrison, & Bombay, 2003; Dijak & Thomspon, 2000). Likewise, at our study sites, forest edge habitat was abundant because water quality improvement was the primary objective of the habitat restoration program that created them. Additionally, nest video has confirmed that these nest predator groups are the primary nest predators at our study sites (40% of 15 documented predation events). And so, it may be that evolutionary fixed habitat preferences potentially associated with nest predator avoidance continue to yield a fitness benefit for this species despite significant anthropogenic changes to land cover in the landscape surrounding these sites.

Although we found that increased understory density and aversion to forest cover within 1,200 m was associated with selection of nest sites and increased nest survival for both species and flycatchers, respectively, there was no concurrent increase in the number of young fledged from successful nests. Indeed, selection for habitat features at all scales yielded no benefit to fledgling production from successful nests. This may be because, in the absence of nest predation, fledgling production is primarily influenced by food availability and microclimate (Martin et al., 2017; Rotenberry & Wiens, 1998). It may be that for both species, there were not sufficient differences in food resources or microclimate among nest sites in our study system and their strategy for nest‐site selection is primarily focused on reducing predation risk. Interestingly, we found fledgling production from successful Bell's Vireo nests was most affected by brood parasitism with parasitized nests producing 1.59 fewer fledglings per successful nest. While this affected only a small proportion of successful nests for this species (4%), it could have population‐level effects when combined with high rates of nest failure due to both higher predation in parasitized nests and parasitism‐related nest abandonment.

Selection for increasing restored habitat and larger patches by vireos may be based on a hierarchical process as suggested by Johnson (1980) whereby choices at different scales may represent trade‐offs between alternative life‐history traits such as adult survival and fitness. For example, second‐order selection (i.e., choice of landscape to breed) may focus on finding a landscape with appropriate resources and potential nesting habitats (Fuller, 2012), whereas finer scale selection may focus on finding a safe nest site that minimizes predation risk (Thomson, Forsman, Sardà‐Palomera, & Mönkkönen, 2006). Selection for larger patches and open cover types such as restored habitat may have evolved under historical conditions and may be based on innate behaviors (Clark & Shutler, 1999) such that individual habitat selection decisions may be formed by evolutionary fixed habitat preferences (Chalfoun & Schmidt, 2012) that historically provided an adaptive advantage. However, in agriculturally fragmented landscapes where nest parasites and predators are concentrated (Batáry & Báldi, 2004), choice of these features may no longer provide a strong adaptive advantage. Importantly, selection for restored habitat was not maladaptive (Kokko & Sutherland, 2001; Schlaepfer et al., 2002; Robertson & Hutto, 2006; Joos, 2013. Alternatively, selection for surrounding restored habitat may provide a fitness benefit not evaluated in this study such as pairing success (Habib, Bayne, & Boutin, 2007), extra‐pair paternity (Biagolini, Westneat, & Francisco, 2017), adult or fledgling survival (Bayne & Hobson, 2002; Streby, Refsnider, & Anderson, 2014, respectively), or could be the result of conspecific attraction (Ward & Schlossberg, 2004).

Beyond broader implications for understanding relationships between habitat selection and reproductive performance, these results have implications for conservation and management for our focal species. Shrubland birds have declined at national, regional, and state levels (Herkert, 1995; Sauer, Hines, & Fallon, 2011), and the extent of these declines for our focal species has led to them being listed as species of conservation concern (USFWS, 2008). To attract increased numbers of both species, land managers should focus on creating or maintaining grassy patches with patches of thick shrubs (e.g., *Cornus* or *Salix* spp.) or young hardwood trees embedded in landscapes with increased open vegetation and limited forest cover. Importantly, to reduce the threat of cowbird parasitism for both species managers should avoid creating habitat patches with increasing cropland cover where cowbirds concentrate (B. Reiley, unpublished). To increase nest success and fledgling production for both species, managers should provide increased opportunities for safe nests sites by maintaining dense vegetation.

To summarize, our study demonstrates that for two species, fine‐scale habitat selection decisions appear to match fitness outcomes, whereas habitat selection at broader scales only provided a fitness benefit for flycatchers. Importantly, even though larger patches and increased restored habitat cover surrounding fields did not lead to enhanced reproductive output, these large‐scale features were associated with increased density of nesting birds. When combined with suitably dense nesting habitat, larger patches of habitat in landscapes with significant grass cover will produce more fledglings per unit area than smaller patches in less grassy landscapes which could help inform conservation decisions that aid in the recovery of these at‐risk species. While the current study focused on limited measures of fitness, inclusion of a broader set of fitness measures such as adult survival (Chalfoun & Schmidt, 2012), fledgling survival (Streby et al., 2014), or obtaining data on individual traits that indicate individual quality, such as lifetime reproductive success (Germain & Arcese, 2014), may elucidate whether habitat preferences that appear neutral, nonideal, or maladaptive based on one measure of fitness may actually be adaptive based on an alternative measure.

## CONFLICT OF INTEREST

The authors declare they have no conflicting interests with the work herein.

## AUTHORS' CONTRIBUTIONS

B.M.R. conceived the ideas, designed methodology, collected the data, and wrote the manuscript; B.M.R and T.J.B. analyzed the data. All authors contributed critically to the drafts and gave final approval for publication.

## Data Availability

The authors intend to archive the data used in this manuscript through the Illinois Data Bank provided by the University of Illinois at Urbana/Champaign.
