# Supplementary material for: Avian fitness consequences match habitat selection at the nest‐site and landscape scale in agriculturally fragmented landscapes
Source: Ecol Evol. 2019 Jun 11;9(12):7173–83. doi: 10.1002/ece3.5288 (PMC6662257; doi:10.1002/ece3.5288)
Supplement: Supplementary file 1 [file ECE3-9-7173-s001.docx]

**AppendixS1**

**Methods**

*Study sites*

We examined fields restored through CREP, a private-land program that is part of the Conservation Reserve Program (CRP), a program known to provide habitat for a variety of conservation priority grassland and shrubland bird species (Pabian et al. 2013). CREP is a voluntary program that provides landowners with a financial incentive to turn cropland into some type of natural perennial vegetation. We surveyed for birds at 172 CREP fields established between 1999 and 2012 in 10 counties in central and west-central Illinois (Figure 1). In this area most remaining natural land cover is forest (~16%) (Walk et al. 2010a). We randomly chose sample fields from 10,818 total fields enrolled in Illinois stratified by county, conservation practice, and size. Focal counties were chosen based on abundance of CREP enrollments and their relative proximity to one another for logistical efficiency. We chose sample fields from 4 out of 11 possible conservation practices (CP22: riparian buffer, CP23: wetland restoration, CP3A: hardwood tree planting, CP4D: permanent wildlife habitat) because these practices were most abundant within the focal counties. Importantly, while fields comprised different conservation practices, habitat structure wasn’t conservation practice specific and so we pooled fields for our analyses. Additionally, we chose fields ≥3.5 ha so that sample locations within fields would be ≥70 m from habitat edges to minimize sampling of birds outside of the focal field (Ralph et al. 1995) and as a result it was very rare for observations to extend beyond the boundary of the field being sampled. Sample fields were comprised of early successional vegetative communities with structure ranging from grasslands consisting of warm and cool season grasses and forb cover to dense woody habitat dominated by shrubs and hardwood trees (*Quercus* spp. or naturally colonizing tree species) and were often adjacent to riparian forests, row-crop agriculture, and pastures.

*Point counts*

Prior to data collection, observers calibrated distances using a laser range finder based on surrounding features. Surveys were conducted between sunrise and 1100 CDT and were not conducted in inclement weather (rain, wind >13mph) (Ralph, Sauer, & Droege, 1993). We used ArcGIS (ESRI 2012) to randomly choose point count locations. Point count locations were > 70 m away from patch edges. We conducted point count surveys between 1-4 times in 2012 (.39, .19, .19, .23, respectively) and 3 times per season in 2013-2015; visits were separated by >14 days. Surveys were conducted by 3 observers in 2012 and 2013 and 4 observers in 2014 and 2015. We rotated observers among patches in all years to minimize potential observer bias and trained observers in species identification and distance estimation prior to data collection.

*Nest searches*

Survey transects consisted of both passive listening and active playback using male vireo and flycatcher vocalizations; these systematic surveys were conducted along a grid of transects at 100-m intervals within each nest sampling field. The number of sample points per field varied depending on patch area and shape. Once per season, observers listened for focal species passively for 2 minutes and then used active playback for 2 minutes with a two speaker CD player set to a volume such that observers could hear broadcasts up to distances of 70 m in moderately dense vegetation with clear atmospheric conditions. All occupied locations and the number of individuals heard singing were documented with GPS coordinates and were used to guide subsequent nest searches.

We recorded GPS coordinates for each nest and monitored nests at 1-4 day intervals to determine status (i.e., active or inactive based on presence of viable eggs or young). We attempted to minimize disturbance to the surrounding vegetation by using different paths to approach and leave the nest site on each visit (Martin & Geupel, 1993).

*Nest-scale variables*

Specifically, within a 5-m radius we estimated the percent cover of grass, forbs, shrubs, and trees within quadrants oriented based on the four cardinal directions. To assess understory vegetation density we used a Robel pole (Robel, Briggs, Dutton, & Hurlburt, 1970) marked at 0.1 m increments and recorded the percent cover of all vegetation in each of four 0.5 m segments from 5 m away in the four cardinal directions at a height of 1 m above the ground. For each vegetation variable, we averaged values from the four cardinal directions into one value.

*Patch size and landscape variables*

Because nests may have higher nest predation and parasitism rates in smaller patches with higher edge to area ratio (Lahti, 2001; Benson, Chiavacci, & Ward, 2013, respectively) we chose to focus on habitat selection relative to patch size. Fields were generally embedded on existing farms and frequently adjacent to riparian areas, roads, and row crop agriculture and so we quantified patch size by measuring all continuous private land habitat around each point count location considering any forest, agricultural fields, and two and four lane roads with disturbed roadsides or habitat transitions (i.e. grassy field to forest interface, etc.) to be field edges. When patches of similar habitat were linked with corridors ≥20 m wide we considered them to be part of the same patch. All patch delineations were ground truthed for accuracy.

Landscape composition around each nest and sample patch was quantified using data from the 2014 National Agricultural Statistics Service Cropland Data Layer (NASS, CDL: Boryan et al., 2014) in ArcGIS 10.1 (ESRI 2012). We used the Geospatial Modeling Environment (Beyer, 2012) to quantify proportions of each cover type within a 200-m buffer around each nest site which was previously demonstrated to be an important scale for early successional birds (Pabian, Wilson, & Brittingham, 2013; Quinn, Johnson, &Brandle, 2014) and for our focal species (Reiley 2017).

**Statistical analyses**

*AIC*

We evaluated single-variable models and selected the highest-ranked model using Akaike’s Information Criterion adjusted for small sample sizes (AIC*_c_*) (Burnham & Anderson, 2002). We assessed support for nest-site habitat variables using model coefficients and their 95% confidence intervals and considered variables that were supported by AIC*_c_*, but for which confidence intervals of coefficients that overlapped zero to be weakly supported.

In addition to ranking candidate models using AIC_c_, we assessed model fit using a likelihood ratio test between the global and intercept-only models, and used the ratio of the chi-squared statistic to degrees of freedom to evaluate evidence of overdispersion (ĉ ≤ 1.2). Support for patch and landscape habitat variables were evaluated using model coefficients and their 95% confidence intervals. We considered variables that were supported by AIC*_c_*, but for which confidence intervals of coefficients that overlapped zero to be weakly supported.

*Detection probability*

Detection probability in our model relied on the distance-sampling likelihood for point transect data (Buckland, Burnham, Anderson, Laake, Borchers, & Thomas, 2001). We fit models evaluating combinations of uniform, exponential, half-normal, and hazard-rate base functions for detection, and evaluated year as a covariate. Candidate models for detection function were evaluated using AIC (Burnham & Anderson, 2002). We right truncated where detection probability was <10% (e.g. Buckland, Burnham, Anderson, Laake, Borchers, & Thomas, 2001) and as a result we only included observations within 100 m, binned into 20 m intervals.

**Literature cited**

Benson, T. J., Chiavacci, S. J., & Ward, M. P. (2013) Patch size and edge proximity are useful predictors of brood parasitism but not nest survival of grassland birds. *Ecological Applications*, 23, 879–887.

Beyer, H. L. (2012) Geospatial Modelling Environment (Version 0.7.2.1). URL: http://www.spatialecology.com/gme. Accessed 25 September, 2014.

Boryan, C., Yang, Z., Di, L., & Hunter, K. (2014) A new automatic stratification method for U.S. agricultural area sampling frame construction based on the Cropland Data Layer. pp. 1939–1404, May 2014, DOI: 10.1109/JSTARS.2014.2322584.

Buckland, S. T., Burnham, D. R, Anderson, K. P.,. Laake, J. L. Borchers, D. L., & Thomas, L. (2001) Distance Sampling: Estimating Abundance of Biological Populations. Chapman & Hall, London.

Burnham, K. P., & Anderson, D. R. (2002) Model selection and multimodel inference: a practical information-theoretic approach. Second edition. Springer-Verlag, New York, New York, USA.

ESRI. 2012. ArcGIS version 10.1. Earth Systems Research Institute, Redlands, California, USA.

Lahti, D.C. 2001.The “edge effect on nest predation” hypothesis after 20 years. *Biological Conservation*, 99, 365–374.

Martin, T. E., & Geupel, G. R. (1993) Nest monitoring plots: methods for locating nests and monitoring success. *Journal of Field Ornithology*, 64, 507–519.

Pabian, S. E., Wilson, A. M., & Brittingham, M. C. (2013) Mixed response of farmland birds to the conservation reserve enhancement program in Pennsylvania. *Journal of Wildlife Management*, 77, 616–625.

Quinn, J. E., Johnson, R. J., & Brandle, J. R. (2014) Identifying opportunities for conservation in embedded in cropland anthromes. *Landscape* *Ecology*, 29, 1811–1819.

Ralph, C. J., Sauer, J. R., & Droege, S. (1993) Managing and monitoring bird populations using point counts: standards and applications. USDA Forest Service General Technical Report PSW-GTR-149. Pacific Southwest Research Station, USDA Forest Service, Albany,CA, USA.Ralph, C. J., Sauer, J. R., & Droege, S. (1995) Monitoring bird populations by point count. USDA Forest Service General Technical Report PSW-149.

Reiley, B. M. (2017) Habitat use, population size, and nesting ecology of conservation priority bird species using restored fields in agricultural landscapes. PhD thesis, University of Illinois, Champaign, IL.

Robel, R. J., Briggs, J. N., Dutton, A. D., & Hurlburt, L. C. (1970) Relationships between visual obstruction measurements and weight of grassland vegetation. *Journal of Range Management*, 23, 295–297.

Table S1. Model selection results for nest-site selection of Bell’s Vireos (*n*=572) and Willow Flycatchers (*n*=132) in restored farmland habitats in western Illinois, USA, 2012–2015.

| Model | K | AIC*c* | ΔAIC*c* | *w_i_* |
| --- | --- | --- | --- | --- |
| *Bell's Vireo* |  |  |  |  |
| % Understory density | **3** | **1227.83** | **0.00** | **1.00** |
| % Shrub | 3 | 1565.25 | 337.42 | 0.00 |
| % Grass | 3 | 1583.62 | 355.79 | 0.00 |
| Constant | 3 | 1589.31 | 361.48 | 0.00 |
| % Forb | 3 | 1590.99 | 363.16 | 0.00 |
| *Willow Flycatcher* |  |  |  |  |
| % Understory density | **3** | **319.71** | **0.00** | **1.00** |
| Constant | 3 | 370.75 | 51.04 | 0.00 |
| % Forb | 3 | 371.59 | 51.88 | 0.00 |
| % Grass | 3 | 372.41 | 52.70 | 0.00 |
| % Shrub | 3 | 372.76 | 53.05 | 0.00 |

Table S2. Number of parameters, ΔAIC*_c_* values, and model weights for all patch and landscape models used to evaluate habitat selection for Bell’s Vireo and Willow Flycatcher in restored farmland patches (*n*=172) in western Illinois, USA, 2012–2015.

|  | Bell's Vireo | | | | Willow Flycatcher | | | |
| --- | --- | --- | --- | --- | --- | --- | --- | --- |
| Model | K | AICc | ΔAIC*_C_* | *w_i_* | K | AIC_c_ | ΔAIC*_c_* | *w_i_* |
| **Landscape models** |  |  |  |  |  |  |  |  |
| Constant | 5 | 3276.92 | 5.08 | 0.04 | 5 | 4585.13 | 21.46 | 0.00 |
| Year | 6 | 3278.95 | 7.11 | 0.01 | 6 | 4585.62 | 21.95 | 0.00 |
| % Grassland within 1200 m | **7** | 3277.88 | 6.04 | 0.02 | 7 | 4585.49 | 21.82 | 0.00 |
| % Forest within 1200 m | 7 | 3278.10 | 6.26 | 0.02 | 7 | 4573.35 | 9.68 | 0.01 |
| % Restored habitat within 1200 m | 7 | 3278.85 | 0.72 | 0.32 | 7 | 4577.34 | 13.67 | 0.00 |
| % Forest + % grass | 8 | 3278.05 | 6.22 | 0.02 | 8 | 4574.46 | 10.79 | 0.00 |
| % Restored habitat + % forest | **8** | **3277.88** | **0.00** | **0.46** | **8** | **4563.69** | **0.00** | **0.99** |
| % Restored habitat + % grass | 8 | 3274.60 | 2.76 | 0.11 | 8 | 4579.04 | 15.37 | 0.00 |
|  |  |  |  |  |  |  |  |  |
| **Patch Models** |  |  |  |  |  |  |  |  |
| Constant | 5 | 3276.92 | 1.39 | 0.30 | **5** | **4585.13** | **0.00** | **0.47** |
| Year | 6 | 3278.95 | 3.42 | 0.11 | 6 | 4585.62 | 0.49 | 0.37 |
| Patch size | **7** | **3275.53** | **0.00** | **0.60** | 7 | 4587.30 | 2.17 | 0.16 |

Table S3. Model selection results of temporal and biological factors related to nest survival of Bell’s Vireo and Willow Flycatcher nests at restored farmland habitats in western Illinois, USA, 2012–2015.

| Models | K | AIC*c* | ΔAIC*c* | *wi* |
| --- | --- | --- | --- | --- |
| *Bell's Vireo* |  |  |  |  |
| Parasitism^a^ | **3** | **1491.49** | **0.00** | **1.00** |
| Nest Stage × Day of Year + Year | 8 | 1527.99 | 36.50 | 0.00 |
| Nest Stage + Year | 7 | 1529.34 | 37.85 | 0.00 |
| Day of Year | 2 | 1534.80 | 43.31 | 0.00 |
| Year × Day of Year | 5 | 1535.20 | 43.71 | 0.00 |
| Constant | 1 | 1541.68 | 50.19 | 0.00 |
| Year | 4 | 1542.15 | 50.66 | 0.00 |
| *Willow Flycatcher* |  |  |  |  |
| Constant | **1** | **429.63** | **0.00** | **0.38** |
| Day of Year | 2 | 429.91 | 0.28 | 0.33 |
| Parasitism | 3 | 431.63 | 2.00 | 0.14 |
| Year | 4 | 432.93 | 3.30 | 0.07 |
| Year × Day of Year | 5 | 433.38 | 3.75 | 0.06 |
| Nest Stage + Year | 7 | 436.93 | 7.30 | 0.01 |
| Nest Stage × Day of Year + Year | 8 | 437.22 | 7.59 | 0.01 |

^a^Probability of Brown-headed Cowbird parasitism.

Table S4. Model selection results of temporal and biological factors related to per nest fledgling production of Bell’s Vireo and Willow Flycatcher nests at restored farmland habitats in western Illinois, USA, 2012–2015.

| Models | K | AIC*c* | ΔAIC*c* | *wi* |
| --- | --- | --- | --- | --- |
| *Bell's Vireo* |  |  |  |  |
| Parasitism^a^ | **3** | **570.68** | **0.00** | **0.97** |
| Constant | 1 | 578.93 | 8.25 | 0.02 |
| Day of Year | 2 | 579.29 | 8.61 | 0.01 |
| Year × Day of Year | 5 | 583.00 | 12.32 | 0.00 |
| *Willow Flycatcher* |  |  |  |  |
| Constant | **1** | **334.72** | **0.00** | **0.46** |
| Day of Year | 2 | 336.32 | 1.60 | 0.21 |
| Parasitism | 3 | 336.70 | 1.98 | 0.17 |
| Year × Day of Year | 5 | 339.35 | 4.63 | 0.05 |

^a^Probability of Brown-headed Cowbird parasitism.
